# Social justice and out‐of‐school science learning: Exploring equity in science television, science clubs and maker spaces

**DOI:** 10.1002/sce.21288

**Published:** 2017-05-29

**Authors:** Emily Dawson

**Affiliations:** ^1^ Science & Technology Studies University College London London WC1E 6BT UK

We cannot take access to equitable out‐of‐school science learning for granted. Data compiled in 2012 show that between a fifth (22% in Brazil) and half (52% in China and the United States) of people in China, Japan, South Korea, India, Malaysia, the United States, the European Union, and Brazil visited zoos, aquaria, and science museums (National Science Foundation, 2012). But research suggests participation in out‐of‐school science learning is far from equitable and is marked by advantage, not least the social axes of age, social class, and ethnicity (Dawson, 2014a, 2014b; National Science Foundation, 2012; OECD, 2012). For instance, in the UK data suggest that the two‐thirds of the population who took part in out‐of‐school science learning activities^1^ in the previous year were more affluent (upper and middle classes) and from the White ethnic majority (Ipsos MORI, 2014). If we believe that out‐of‐school science learning provides valuable educational, cultural, social and political opportunities, then we must take questions of equity seriously.

Ideas from social justice can help us understand how equity issues are woven through out‐of‐school science learning practices. In this paper, I outline how social justice theories, in combination with the concepts of infrastructure access, literacies and community acceptance, can be used to think about equity in out‐of‐school science learning. I apply these ideas to out‐of‐school science learning via television, science clubs and maker spaces, looking at research as well as illustrative examples to see how equity challenges are being addressed in practice. I argue that out‐of‐school science learning practices can be understood on a spectrum from weak to strong models of social justice. Thinking about social justice as a spectrum helps us think through what equitable out‐of‐school science learning practices might involve, both to analyze existing practices and, importantly, to imagine new, more inclusive ones.

Out‐of‐school science learning is a broad term, used to describe quite different activities, participants, aims, and practices. It can mean enjoying science festivals, watching science documentaries, pursing science‐related hobbies as well as activities focused on engineering, mathematics, or technology (see, e.g., Bonney et al., 2009; Dingwall & Aldridge, 2006; Kaiser, Durant, Levenson, Wiehe, & Linett, 2013). In this paper, I focus primarily on the contrasting worlds of television and science clubs as out‐of‐school science learning contexts^2^. I use “science” as an umbrella term for science, technology, engineering, or mathematics related subjects. However, I add a caveat to how I use the term out‐of‐school. Because “out‐of‐school” invokes the idea of school, there can be a tendency to focus on youth as participants and activities that are for, by, or with youth. But of course adults may not consider their television watching an “out‐of‐school” activity. Thus, I note here that I keep both adults and youth in mind when writing about equity and out‐of‐school science learning.

## 1 UNDERSTANDING EQUITY FOR OUT‐OF‐SCHOOL SCIENCE LEARNING

Many discussions of equity and out‐of‐school learning have presented equity issues primarily in terms of access and barriers to access (Charlton et al., 2010; Dawson, 2014c; Institute of Physics, 2014). The danger here is of framing equity in out‐of‐school science learning as a kind of crusade (Dawson, 2014c). That is, exposing more people to science is *de facto* a good thing, whether they want it or not, an assumption Lee and Buxton describe as assimilationist (2010). While science certainly has many benefits, such perspectives belie the potential for damage caused by science and science learning practices that have been called out as colonialist, racist, misogynistic, heteronormative, or otherwise oppressive (see, for example, Cassidy, Lock, & Voss, 2016; Harding, 2008; Medin & Bang, 2014; Pollock & Subramaniam, 2016). How then can we think about equity in ways that goes beyond assimilation in science and science learning? In what follows I outline two theories of social justice and build on them to show how the concepts of infrastructure access, literacies, and community acceptance can be used to understand equity along a spectrum of weak to strong socially just practice.

Social justice theorists have long been concerned with how resources might be distributed most equally (redistributive social justice), and, more recently, most equitably (relational social justice). In the first model, justice is about equality of access and distribution between social groups; everybody being able to do, enjoy or use the same amounts of the same things (Rawls, 1971). The second model, in contrast, emphasizes the value of recognizing difference. That is, recognizing, respecting, and valuing that people differ and taking their differences into account, rather than treating everyone's needs as the same (Young, 1990).

Of course, redistributive and relational models of social justice need not sit in opposition to each other. Indeed, as Fraser (2003) has argued, combining both models of social justice provides us with a powerful tool for thinking about and addressing issues of inclusion/exclusion. Such an approach requires a commitment to exploring beyond issues of access and participation (weak inclusion) to include questions of knowledge, representation, power, and cultural change (strong inclusion). For instance, if we apply these ideas to scientific practice, scholars have argued that it is not enough to recruit more ethnically diverse scientists, more female scientists, or more scientists from working class backgrounds, without simultaneously changing the culture and content of scientific knowledge (Harding, 2008; Longino, 1990; Schiebinger, 2007). Without both pieces of the puzzle, science practices will struggle to become more inclusive.

The combination of redistributive and relational social justice is the basis I use for framing equity and inclusion in out‐of‐school science learning using the concepts of infrastructure access, literacies, and community acceptance along a spectrum (Dawson, 2014a; Grabill, 1998; Porter, 1998). These three concepts serve as lenses, or levels of analysis, for understanding what might change and can be understood in weak and strong forms. Weak infrastructure access is about the extent to which people are able to access a field and the institutions, resources, or practices within it (drawing only on redistributive social justice). Examples of activities based on weak infrastructure access include the many “women into science” programs much criticized by scholars for attempting to change women rather than changing practices and cultures within the scientific community to welcome, respect, and represent women (Phipps, 2008; Schiebinger, 2007). In contrast, strong infrastructure access encompasses both physical access *and* the extent to which people have power to shape those spaces and activities to fit their needs, drawing on both redistributive and relational social justice.

The concept of literacies highlights the multiple, often hidden, literacies required to be able to participate in out‐of‐school science learning practices (Dawson, 2014a). For instance, in monolingual science clubs you may need to know the actual language used, a degree of scientific literacy as well as practical “know‐how” (such as how to use specific tools) in order to be able to learn science. A weak reading of this concept focuses on surfacing the literacies that facilitate access to out‐of‐school science learning, and supporting participants to develop the literacies they need (in other words, to change themselves). In contrast, the strong interpretation of literacies involves critical literacy and thinking about power (Delpit, 1988). For instance, whose selves, knowledges, languages, and ways of being are recognized, represented, and welcomed in out‐of‐school science learning practices and how might these be resisted or opened up? This stronger form of social justice has implications for changes in practice, institutions, and policies, rather than only changing participants.

Finally, community acceptance can also be employed in a dual sense. First, to think about how existing stakeholders, or “insiders”, involved in out‐of‐school science learning welcome new participants and change their practices to do so (Porter, 1998). Second, to understand the views, experiences, and expectations of marginalized groups about whether opportunities seem relevant and valuable. Importantly, it makes space for participants to reject as well as be excluded from science learning practices (Dawson, 2014a, 2014b). As above, considering both “insiders” and “outsiders” constitutes a stronger version of community acceptance than focusing only on one or the other. Thinking about infrastructure access, literacies, and community acceptance as conceptual lenses or levels of analysis is helpful because they highlight the multifaceted nature of equity issues and the cumulative effect of addressing multiple issues. In using these concepts on a spectrum of weak to strong forms of social justice, I do not mean to imply that weaker versions of equity are not important, often they are fundamental; however, they are rarely sufficient. Instead, thinking about social justice as a spectrum helps to foreground multiple perspectives and the importance of both redistributive and relational social justice in thinking through equity in out‐of‐school science learning.

## 2 EXAMINING EQUITY IN TWO OUT‐OF‐SCHOOL SCIENCE LEARNING CONTEXTS

### 2.1 Science television

Research shows that television remains a key form of engagement with science for many people and can represent a significant site for science learning (Dhingra, 2006; Miller, Augenbraun, Schulhof, & Kimmel, 2006). For instance, a survey in the UK found that 59% of adults saw television as their primary source of scientific information (Ipsos MORI, 2014). Examining equity concerns about science on television however, raises several questions. In what follows, I briefly explore equity issues in terms of professional attitudes, content, and representation and, finally, what audiences make of science television using the concepts of infrastructure access, literacies and community acceptance.

Research suggests that, for science television, professional infrastructure access and “insider” community acceptance are limited two key ways, first in terms of who can access a television career and second in terms of audiences. Within the television industry in the UK, for instance, equity issues have been translated into goals around diversifying the workforce. As a result, the industry regularly reproduces documents (or diversity charters) about goals for equitable practice (see for example BBC, 2016; Channel 4, 2015). Even if more inclusive recruitment practices are enacted as a result of these charters, this tactic remains at best a weak form of infrastructure access, one focused on training diverse staff rather than changing exclusive work practices. As such, the relational aspects of social justice are eclipsed by the redistributive. At worst, such documents may work to obscure the lack of change in practice (cf. Ahmed, 2012). For instance, television production is a notoriously difficult career pathway for people who are not White, male, or relatively wealthy (Dent, 2016; Oakley & O'Brien, 2016). Research on who makes television therefore calls into question the extent to which even weak professional infrastructure access is enacted, which suggests “insider” community acceptance of new, more diverse colleagues is limited.

Audiences for science television are framed in similarly limited ways within the industry. In research with science television producers the market logic of viewing figures governed how equity and inclusion were framed (Dawson, Seakins, Archer, Calabrese Barton, & Dierking, 2015). Indeed, using viewing figures as the only important measure of participation reinforced the view that television is first and foremost a business (Florensa, Hochadel, & Tabernero, 2014). Viewing figures were understood as the key measure of success for science television producers and their commissioners. Higher viewing figures were assumed to include more people who might be considered underserved or disadvantaged, thus negating any need to tailor content or production processes to be more inclusive, a very weak form of infrastructure access (Dawson et al., 2015).

If we look at television content, or how science stories and people on television are represented, research on gender illustrates important equity issues. Studies of how youth identified with scientists when watching television revealed that, as might be expected, boys tended to identify with male scientists while girls tended to identify with female scientists (Steinke, Applegate, Lapinski, Ryan, & Long, 2012). Placed alongside other research on children's science television, this finding takes on a more unsettling implication. Research on young people's viewing habits found White male scientists were significantly overrepresented in children's programs about science, technology, mathematics, and engineering, across factual, drama, and cartoon shows (Whitelegg, Holliman, Carr, Scanlon, & Hodgson, 2008). Indeed, looking beyond children's television, research suggests that people are represented in television science stories in ways that reproduce structural inequalities such as gender, class and ethnicity (among others) (Chimba & Kitzinger, 2010; Fisher & Cottingham, 2016; Flicker, 2003). As such the representation of science on television follows normative structures about science narratives as White, male dominated stories (McNeil, 2007). The content of science television can therefore be considered as following a weak form of critical literacy in terms of limited representation, respect for difference, and relational social justice.

In contrast, a stronger approach to literacies and social justice involves representing and valuing a more pluralistic view of what science is and how people are involved with science. To this end, some interesting examples of equitable practice can be drawn on in science television programs that have sought to change how science stories are told (cf. Paulsen, 2013). Although such initiatives are usually aimed at youth rather than adults, in explicitly centering equity concerns in their content they disrupt conventional narratives about who can do science. Take, for example, the SciGirls television show developed in the United States (PBS, 2017). The multiplatform program aims specifically at creating female friendly content, representing girls and women from a range of ethnic backgrounds exploring science on television, online, and through educational outreach workshops. The project takes a strong approach to literacy issues through the development of Spanish language episodes as well as addressing critical literacy through content that reflects on power in science (Knight‐Williams, Williams, Teel, Willaims, Hernandez, Negrete & Rahbari, 2016). Projects such as SciGirls are interesting in equity terms since in actively supporting girls to pursue science they disrupt the normative practices of science on television.

Turning finally to audiences suggests further equity challenges. While watching television may be easier than going to a museum, purposefully watching *science* on television may not be a common practice in most households (Bennett et al., 2009; Ipsos MORI, 2014). For instance, longitudinal research with families in the UK showed that watching science television was opportunistic and rarely used to actively support everyday science learning, especially among working class families (Archer et al., 2012). Indeed, decades of research on television and communication theory suggest that audiences reconstruct the meaning of the programs they watch (Morley, 2006; Skeggs & Wood, 2011). Thus, in addition to issues of weak infrastructure access, questions of literacies and audience community acceptance are raised in thinking about science television as an out‐of‐school science learning context. Notably, that we cannot assume that watching television leads to learning about science. For instance, what degree of scientific literacy is needed to turn watching an episode of “Crime Scene Investigation” into a science learning opportunity? Furthermore, doing so requires that those watching science on television see the content as relevant and interesting enough to wish to do so (community acceptance). Exploring how and where weak and strong forms of literacy and audience community acceptance are enacted would provide valuable insights about how science television is used (or not) in out‐of‐school science learning.

### 2.2 Science clubs and maker/hackerspaces

After‐school and community clubs represent another out‐of‐school science learning context where people participate often over long periods of time. Like television, science clubs are often woven into people's lives through frequent and regular participation. Unlike television, clubs have different affordances for power‐sharing and participant‐led activities since the relationship between producers and participants is typically much closer. Since the structure of science clubs differs from that of television, in this section I first explore equity issues in youth science clubs, before examining equity in a specific genre of adult science club, the maker or hackerspace.

Research suggests that science clubs can be empowering spaces for socioeconomically disadvantaged youth to leverage their own knowledge and practices to address science issues relevant to themselves and their communities (Barton & Tan, 2010; National Research Council, 2015). The Austrian Knowledge Rooms provide a useful example of working toward a strong model of equitable practice in a community youth club setting. Organized by the Austrian Science Centre Network (ASCN), the pop‐up Knowledge Rooms use empty shop fronts in Vienna to run science clubs for youth from disadvantaged minority ethnic backgrounds. Importantly, the Knowledge Rooms begin with community consultation, are based inside disadvantaged neighborhoods and work with youth to codevelop the rules and activities of each space, thus enacting a strong approach to infrastructure access. The “rules” are then displayed on the walls to help overcome literacy issues about not knowing what to do in the science club (ASCN, 2017; Streicher, Unterleitner, & Schulze, 2014). While the impetus and resources to set up a Knowledge Room typically comes from the ASCN, limiting power sharing in some sense, practitioners work closely with youth and their families to develop relevant and fun activities. These practices support strong community acceptance in both forms (Streicher et al., 2014).

Studies also suggest that creating clubs based on a strong version of social justice that supports youth empowerment with and through science is far from easy. For example, Rahm's (2010) study of three different science clubs (in Canada and the United States) found socioeconomically disadvantaged youth struggled to identify with science or see science as part of their futures, despite long‐term involvement and youth‐led projects. In two other projects focused on carefully supporting and nurturing science learning in girl‐led science clubs, the girls’ involved still concluded science was largely irrelevant to their lives (Gonsalves, Rahm, & Carvalho, 2013; Thompson, 2014). Notably, the clubs these studies examined employed a strong version of social justice. Participation went beyond getting youth through the club doors, such that youth were central to the planning and implementation of activities, with careful attention paid to ameliorating structural inequalities and empowering youth. Indeed, a study of how practitioners in science youth clubs understood and addressed equity issues found that those involved in community and club settings were the most vocal advocates of equitable practices and most able to provide examples and evidence of what this entailed (Dawson et al., 2015). That is, “insider” community acceptance, as well as infrastructure access and literacies appeared in a strong form. Thus, it is particularly notable that “outsider” community acceptance on the part of participating youth was still somewhat limited even in youth science clubs that took a strong approach to social justice. Despite their involvement in these out‐of‐school science learning settings, youth participants still struggled to see themselves within science.

Turning to science clubs and community groups with adult participants shows another side to these out‐of‐school science learning contexts. Adult science clubs are typically the preserve of amateur enthusiasts. As a result, participants are usually very knowledgeable and strongly motivated about their area of interest (Azevedo, 2011). Nonetheless, equity issues mark these settings. Take, for example, maker or hackerspaces. These community‐led science clubs, based on a German model of open spaces where people gather to adapt, play with or otherwise creatively hack technology and pursue engineering projects, have been criticized as spaces of White, male privilege, open in name only (Fox, Ulgado, & Rosner, 2015; Willett, 2016).

In response, feminists and people from minority ethnic backgrounds have established their own makerspaces (Maalsen & Perng, 2016; Toupin, 2014). Such clubs aim to provide safe, welcoming spaces for women and/or people from minority ethnic backgrounds through providing tailored support and explicitly valuing their skills and traditions, integrating these into the clubs (Rosner, 2014). For instance, a U.S. hackerspace in Berkeley, California, is run for mothers, by mothers, combining tech, crafts, workshop sessions, alongside the all important childcare that underpins whether participation is possible (Hackermoms, 2017; Rosner & Fox, 2016). Similarly, in the Dublin PyLadies club, set up in 2013 explicitly to counteract the male‐dominated landscape of computer programming, women meet monthly to socialize, code together, and network with industry (Maalsen & Perng, 2016).

In one sense, these clubs take a strong approach to social justice. They take structural inequalities into account and transform practices to support strong forms of infrastructure access (both in terms of access and power sharing), including implicit critical literacy issues that support the rightful presence of a more diverse group of hackers, fixers, or makers. There is, however, a significant tension between openness in making/hacking, on the one hand, and protective support for women, mothers, or people otherwise excluded from other makerspaces, on the other hand, as noted in the emerging scholarship (Fox et al., 2015; Nascimento, 2014; Rosner & Fox, 2016; Toupin, 2014). An out‐of‐school science learning landscape marked by segregation, whether based on gender, ethnicity, or another aspect of our selves, still presents serious challenges to social justice. Thus, while infrastructure access, literacies, and community acceptance in feminist clubs may be strong, questions remain about how equity is understood and enacted.

The tensions involved in learning environments tailored specifically to one group or another have of course been much discussed (cf. Forde, 2014). Thus, I note here only first, that it is crucial to create safe, welcoming science clubs for youth, women, and/or people from minority ethnic backgrounds where their knowledges and practices can be respected and valued. And second, that in thinking about social justice we must also question the extent to which a separated system of inclusive out‐of‐school science learning clubs can interrogate and transform the wider, more established field of practice of maker spaces or, indeed, of practices in the broader fields of science and technology. Thus, for both adults and youth, while science clubs appear to provide significant opportunities for social justice, they remain constrained by structural inequalities in ways that seem hard to change.

## 3 DEVELOPING EQUITABLE OUT‐OF‐SCHOOL SCIENCE LEARNING

What can we learn from the brief and admittedly partial picture, painted here? This paper raises both troubling and hopeful issues for researchers, practitioners, participants, and policy makers interested in how to understand and work with social justice in out‐of‐school science learning contexts. The analysis presented here suggests equity issues remain a significant challenge for out‐of‐school science learning. While science television, science clubs and maker spaces may be an important site for some youth or adults to enjoy and engage with science, normative social structures about who can *do* science remain problematic and appear to limit strong forms of social justice and inclusive practice. Applying both redistributive and relational social justice to equity in out‐of‐school science learning using the concepts of infrastructure access, literacies and community acceptance as conceptual lenses on a spectrum from weak to strong highlights how complex, multifaceted, and cumulative equity issues are. More hopefully, however, the theoretical framework described here can be used to understand what it is that might make one activity more equitable than another in terms of weak or strong social justice. That is, practitioners, policy makers and researchers can use this framework to think about how we can break down multiple, complex, and overlapping issues to develop more inclusive out‐of‐school science learning practices.

Notably, in both television, science clubs and maker spaces people have taken up the challenge of developing more equitable practices, even if such projects are the exception rather than the norm. Pockets of equitable practice are important because a key challenge in embedding equity in out‐of‐school science learning is rooted in the need for large‐scale social and field‐wide change. For instance, thinking about making out‐of‐school science learning more equitable necessarily includes thinking about inclusion in the fields of science and technology. Change on this scale, within and across out‐of‐school science learning contexts, science education more broadly and, if we are to be ambitious, science and technology writ large, is no mean feat. Each experiment with equitable practice, however small, helps us to embrace this challenge, because as Lorde suggests, “revolution is not a onetime event. It is being always vigilant for the smallest opportunity to make a genuine change in established, outgrown responses” (1984, pp. 140–141). From this perspective, the value of projects such as SciGirls or Knowledge Rooms lies in their hopeful capacity to challenge and resist normative social structures around who can and who cannot take part in out‐of‐school science learning, or science more broadly.

## ENDNOTES

1 The activities on the survey were science festivals, science museums, science centers, zoos and aquaria, botanic gardens, planetariums, nature reserves, science talks, and science activities not involved in school or university education (Ipsos MORI, 2014).

2 The theoretical framework extended in this essay to science television and science clubs was initially developed in relation to museum and science center visiting (Dawson, 2014a).
